# Pharmacokinetics and bioequivalence of a molnupiravir tablet formulation compared with the molnupiravir capsule formulation in healthy adult participants—a randomized, open-label, three-period, crossover study

**DOI:** 10.1128/aac.01434-24

**Published:** 2025-02-06

**Authors:** Julie L. Fiore, Yoon Jin, Tycho Heimbach, Shruti R. Patel, Tian Zhao, Catherine Z. Matthews, Sandra Pagnussat, Brian M. Maas, Mickie H. Cheng, S. Aubrey Stoch

**Affiliations:** 1 Merck & Co., Inc. 2793 , Rahway, New Jersey, USA; 2 QPS, Miami, Florida, USA; IrsiCaixa Institut de Recerca de la Sida, Barcelona, Spain

**Keywords:** bioequivalence, COVID-19, antiviral, pharmacokinetics

## Abstract

Molnupiravir, a prodrug of β-D-N^4^-hydroxycytidine (NHC), is administered orally as four 200 mg capsules twice daily for 5 days to treat COVID-19. This randomized, open-label, four-treatment sequence, three-period crossover study (NCT06615869) evaluated the bioequivalence of a new single 400 mg oral dose of the molnupiravir tablet Formulation 1 (F1) to a 400 mg oral dose of the currently authorized molnupiravir capsule formulation (administered as two 200 mg capsules) by comparing the plasma pharmacokinetics of NHC following administration to healthy participants. The effect of food on the plasma NHC pharmacokinetics following the administration of the molnupiravir F1 tablet, safety and tolerability of a single oral 400 mg dose of molnupiravir, and pharmacokinetics of a separate molnupiravir tablet Formulation 2 (F2) with a slower *in vitro* dissolution rate were also evaluated. The geometric mean ratio and 90% confidence intervals ([1 × 400-mg F1 tablet]/[2 × 200 mg reference capsules]) for plasma NHC area under the concentration-time curve (AUC) from time 0–infinity, AUC from time 0–last measurable time point, and maximum plasma concentration were 1.00 (0.97, 1.03), 1.00 (0.97, 1.03), and 0.98 (0.93, 1.03), respectively. All estimates were within prespecified limits (0.80, 1.25), demonstrating bioequivalence of the molnupiravir F1 tablet and reference capsules. Administration of the F1 tablet with a high-fat meal did not meaningfully impact the rate or extent of absorption. The pharmacokinetics of the F2 tablet were similar to the reference capsules. Administered in either formulation, molnupiravir was generally safe and well tolerated. In conclusion, a single 400 mg tablet of molnupiravir is bioequivalent to the reference capsule formulation in healthy adults.

**CLINICAL TRIALS:**

This study was registered at ClinicalTrials.gov as NCT06615869.

## INTRODUCTION

Molnupiravir is an orally administered isobutyryl ester prodrug of the antiviral nucleoside analog β-D-N^4^-hydroxycytidine (NHC) authorized for emergency use in the treatment of mild-to-moderate coronavirus disease 2019 (COVID-19) in nonhospitalized adults at risk of severe disease (1, 2). Following oral administration, molnupiravir is rapidly absorbed and hydrolyzed by esterases to NHC primarily during absorption and/or hepatic first pass (3); therefore, no significant concentrations of molnupiravir are detected systemically in plasma (4, 5). NHC is distributed systemically and converted intracellularly into its active form, NHC-triphosphate (NHC-TP) (2, 4, 6, 7). NHC-TP acts via viral error induction, causing the accumulation of nucleotide errors in viral RNA with each cycle of replication, ultimately leading to the production of nonviable, noninfectious virus (8–10).

Oral antiviral therapies offer increased convenience over agents administered via intravenous infusion. For the treatment of COVID-19, molnupiravir is administered as oral capsules, with or without food, at a dose of 800 mg (four 200 mg capsules) twice daily for 5 days. The 200 mg capsule formulation was developed to support accelerated access during the COVID-19 pandemic. A smaller, yet qualitatively similar, 400 mg film-coated molnupiravir tablet was later developed to reduce pill burden and improve ease of administration.

The primary aim of this study was to evaluate the bioequivalence of the currently authorized molnupiravir capsule (from here on, termed “reference capsule,” administered as two 200 mg capsules) and the molnupiravir F1 tablet (administered as one 400 mg tablet) by comparing the plasma pharmacokinetics (PK) of NHC. A secondary objective was to establish the effect of a high-fat meal on the PK of plasma NHC following administration of the molnupiravir F1 tablet. An additional secondary objective was to investigate the PK of a separate molnupiravir tablet (Formulation 2, F2, designed with a slower release rate) relative to the reference capsule formulation to provide further clinical data to inform the dissolution bioequivalence safe space (11, 12). We also aimed to evaluate the safety and tolerability of single oral doses of molnupiravir in capsule and tablet formulation.

## RESULTS

### Participants

**Fig 1:**
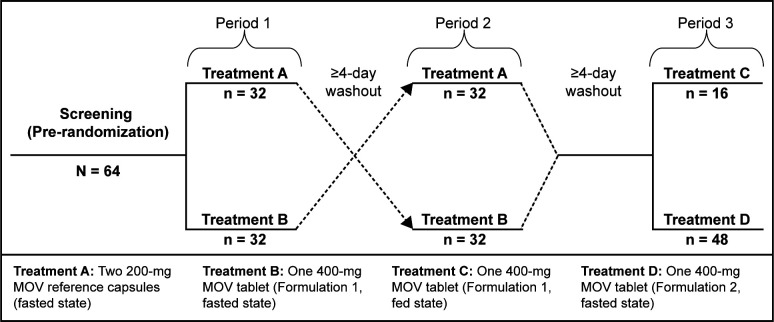
Study design. A total of 64 participants were randomized at study onset to a sequence of Treatments A and B (1:1) in Periods 1 and 2 and then Treatment C or D (1:3) in Period 3. MOV, molnupiravir.

Between 20 January 2023, and 15 March 2023, 64 participants were randomly assigned 4 possible treatment sequences across 3 periods. Thirty-two participants were assigned to receive Treatment A (two 200 mg molnupiravir reference capsules in the fasted state), and 32 were assigned to receive Treatment B (one 400 mg molnupiravir F1 tablet in the fasted state) in Period 1, with Treatments A and B crossover in Period 2; 16 were assigned to receive Treatment C (one 400 mg molnupiravir F1 tablet in the fed state) in Period 3; and 48 were assigned to receive Treatment D (one 400 mg molnupiravir F2 tablet in the fasted state) in Period 3 (Fig. 1). Sixty-three participants completed all treatments and were included in the per-protocol population and the safety population (participants who received ≥1 dose of study intervention), used for primary PK analysis and safety analysis, respectively. One participant discontinued from the study on day 5 after completing Period 1 (Treatment A; more information can be found in the “Safety” section, below).

Baseline characteristics of participants were balanced (Table 1). In the overall population, more than 90% of participants were White. Weight ranged between 50.1 and 99.8 kg. Among these healthy participants, 19 participants (29.7%) reported prior COVID-19 infection. Twelve participants (17.8%) reported having medical conditions (obesity [*n* = 12], dysmenorrhea [*n* = 1], seborrheic dermatitis [*n* = 1], and varicose veins [*n* = 1]). The only prior treatment or medication reported was birth control, which was reported for 13 participants (18.8%). Concomitant treatments or medications were reported for 18 participants; they were reviewed as part of study monitoring, and none were considered clinically relevant within the context of the study. None of the participants reported receiving COVID-19 vaccination in the 14 days before enrollment.

**TABLE 1:** Participant characteristics in the safety population^*a*^

	Treatment A	Treatment B	Treatment C	Treatment D
Participants in population, *n*	64	63	16	47
Sex, *n* (%)				
Male	30 (46.9)	29 (46.0)	6 (37.5)	23 (48.9)
Female	34 (53.1)	34 (54.0)	10 (62.5)	24 (51.1)
Age, years				
Mean (SD)	39.3 (8.8)	39.5 (8.7)	37.4 (8.1)	40.2 (8.9)
Median (range)	40.0 (23–56)	40.0 (23–56)	36.5 (23–50)	41.0 (23–56)
Race, *n* (%)				
Black or African American	5 (7.8)	5 (7.9)	1 (6.3)	4 (8.5)
White	59 (92.2)	58 (92.1)	15 (93.8)	43 (91.5)
Ethnicity, *n* (%)				
Hispanic or Latino	64 (100.0)	63 (100.0)	16 (100.0)	47 (100.0)
Weight, kg				
Participants with data, *n*	64	63	16	47
Mean (SD)	76.47 (11.32)	76.27 (11.30)	75.06 (10.51)	76.68 (11.64)
Median (range)	77.20 (50.1–99.8)	77.10 (50.1–99.8)	77.05 (58.3–93.1)	77.30 (50.1–99.8)
BMI, kg/m²				
Participants with data, *n*	64	63	16	47
Mean (SD)	27.50 (2.36)	27.49 (2.38)	27.71 (1.92)	27.42 (2.53)
Median (range)	27.50 (18.9–32.2)	27.40 (18.9–32.2)	28.10 (24.3–30.2)	27.40 (18.9–32.2)

^*a*^ BMI, body mass index; SD, standard deviation. Treatment A = Two 200 mg molnupiravir reference capsules (fasted state). Treatment B = One 400 mg molnupiravir Formulation 1 tablet (fasted state). Treatment C = One 400 mg molnupiravir Formulation 1 tablet (fed state). Treatment D = One 400 mg molnupiravir Formulation 2 tablet (fasted state).

### Pharmacokinetics

**Fig 2:**
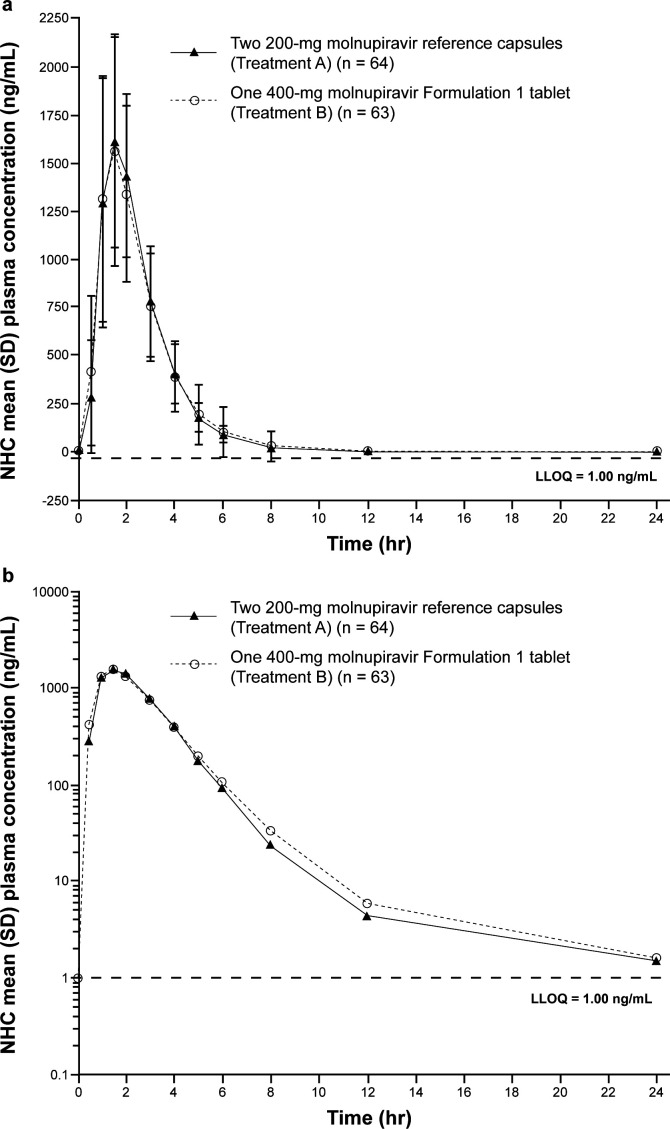
Arithmetic mean (±SD) plasma concentration-versus-time profiles of NHC following administration of two 200 mg molnupiravir reference capsules in the fasted state (Treatment A) and one 400 mg molnupiravir Formulation 1 tablet in the fasted state (Treatment B) to healthy adults. (a) Linear scale, (b) semi-log scale. LLOQ, lower limit of quantitation; NHC, β-D-N4-hydroxycytidine; SD, standard deviation.

The arithmetic mean concentration-versus-time profiles of plasma NHC following administration of the reference capsules and the F1 tablet to healthy adult participants were similar (Fig. 2). The geometric mean ratios (GMRs), reported as F1 tablet/reference capsule (with 90% confidence intervals [CIs]), of plasma NHC area under the concentration-time curve (AUC) from time 0 to infinity (AUC_0–inf_), AUC from time 0 to the last measurable point (AUC_0–last_), and maximum plasma concentration (C_max_) were 1.00 (0.97, 1.03), 1.00 (0.97, 1.03), and 0.98 (0.93, 1.03), respectively (Table 2). All GMR and 90% CIs were within the prespecified limits of 0.80 and 1.25 as per the U.S. Food and Drug Administration (FDA) and European Medicines Agency (EMA) guidelines for bioequivalence (13, 14), thereby demonstrating the bioequivalence of the molnupiravir F1 tablet and molnupiravir reference capsules. Other PK parameters, such as terminal half-life (t_1/2_) and time to maximum concentration (T_max_), were also all found to be similar between the reference capsule and the F1 tablet (Table 2).

**TABLE 2:** Statistical comparison and summary statistics of NHC plasma pharmacokinetics following the administration of two 200 mg molnupiravir reference capsules in the fasted state (Treatment A) and one 400 mg molnupiravir Formulation 1 tablet in the fasted state (Treatment B) to healthy adults^*a*^^,*b*^

Pharmacokinetic parameter	Two 200 mg molnupiravir reference capsules (fasted state)			One 400 mg molnupiravir Formulation 1 tablet (fasted state)			Ratio^*f*^		% CV^*g*^
	N	GM	95% CI	N	GM	95% CI	GMR	90% CI	
AUC_0–inf_ (hr⋅ng/mL)^*c*^	64	4,050	(3,800, 4,310)	63	4,050	(3,810, 4,320)	1.00	(0.97, 1.03)	10.1
AUC_0–last_ (hr⋅ng/mL)^*c*^	64	4,040	(3,790, 4,310)	63	4,050	(3,800, 4,310)	1.00	(0.97, 1.03)	10.1
AUC_0–12_ (hr⋅ng/mL)^*c*^	64	4,010	(3,760, 4,270)	63	4,010	(3,770, 4,270)	1.00	(0.97, 1.03)	10.1
C_max_ (ng/mL)^*c*^	64	1,680	(1,560, 1,820)	63	1,640	(1,510, 1,790)	0.98	(0.93, 1.03)	17.7
T_max_ (hr)^*d*^	64	1.50	(1.00, 3.00)	63	1.50	(1.00, 5.00)	NC	NC	NC
t_½_(hr)^*e*^	64	2.88	71.4	63	3.26	66.9			
CL/F (L/hr)^*e*^	64	77.8	25.8	63	77.5	25.4			
V_z_/F (L/hr)^*e*^	64	323	66.4	63	364	63.8			

^*a*^ AUC_0–12_, area under the concentration-time curve from time 0 to 12 hours; AUC_0–inf_, area under the concentration-time curve from time 0 to infinity; AUC_0–last_, area under the concentration-time curve from time 0 to the last measurable time point; CI, confidence interval; CL/F, apparent plasma clearance; C_max_, maximum plasma concentration; CV, coefficient of variation; GM, geometric mean; GMR, geometric mean ratio; NC, not calculated; NHC, β-D-N4-hydroxycytidine; t_½_, half-life; T_max_, time to maximum plasma concentration; V_z_/F, apparent volume of distribution.

^*b*^ One participant did not participate in Period 2.

^*c*^ Back-transformed least squares mean (mean difference) and confidence intervalCI from linear mixed-effects model performed on natural log-transformed values.

^*d*^ Median (min, max) reported for Tmax.

^*e*^ Geometric mean and percent geometric CV reported for t½, CL/F, and Vz/F.

^*f*^ Reported as one 400- mg molnupiravir Formulation 1 tablet (fasted state) / two 200- mg molnupiravir reference capsules.

^*g*^ Pseudo within-subject % CV = 100 × Sqrt [(σp2 + σq2 – 2 × σpq)/2], where σp2 and σq2 are the estimated variances on the log scale for the 2two treatment groups, and σpq is the corresponding estimated covariance, each obtained from the linear mixed-effects model.

**Fig 3:**
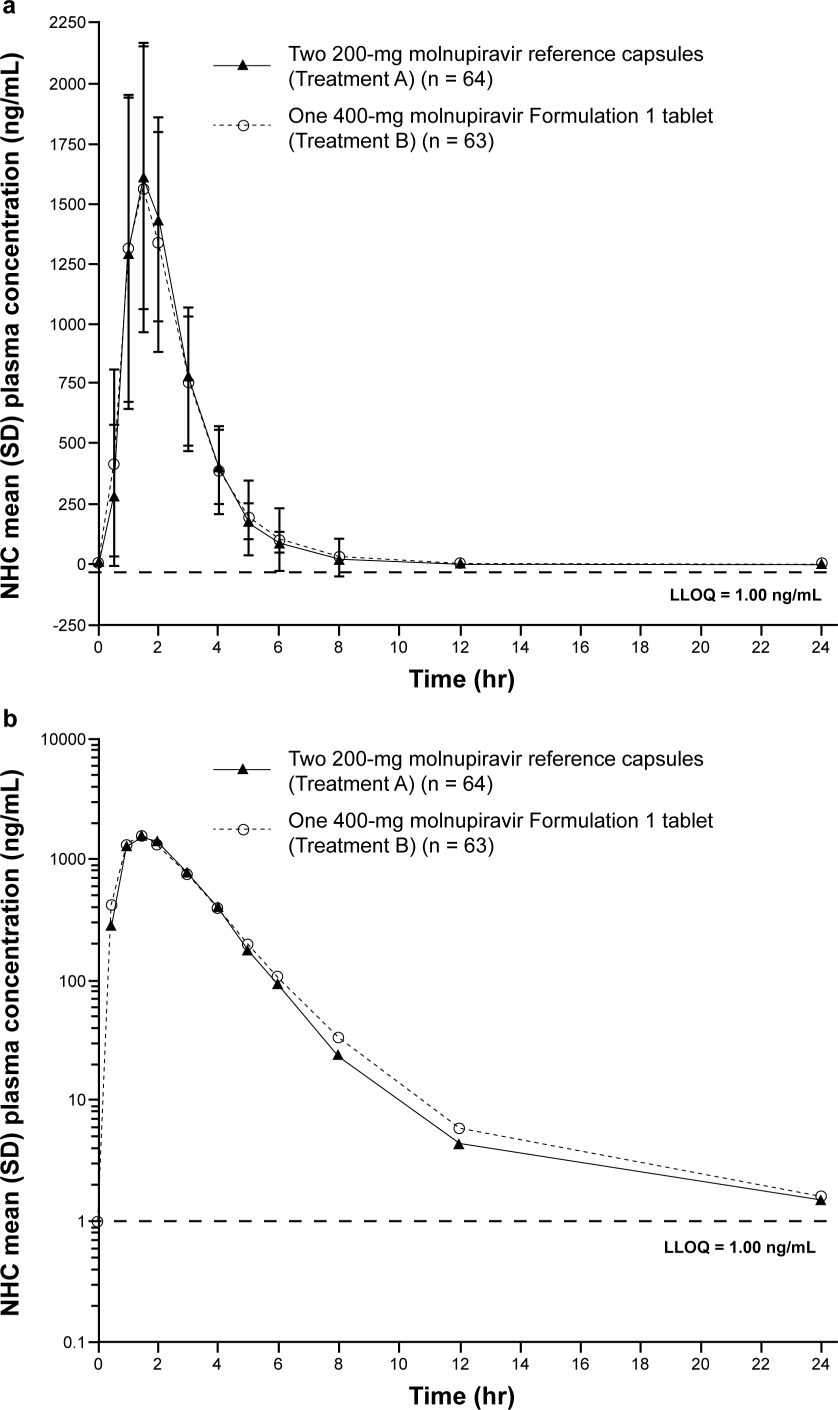
Arithmetic mean (±SD) plasma concentration-versus-time profiles of NHC following administration of one 400 mg molnupiravir Formulation 1 tablet in the fasted state (Treatment B) and in the fed state (Treatment C) in healthy adult participants. (a) Linear scale, (b) semi-log scale. LLOQ, lower limit of quantitation; NHC, β-D-N4-hydroxycytidine; SD, standard deviation.

The effect of a high-fat meal on the plasma PK of NHC following oral administration of the 400-mg F1 tablet was assessed by comparing the plasma PK of NHC in the fed and fasted state. The NHC AUC_0–inf_, AUC_0–last_, and C_max_ GMRs (90% CIs), reported as fed/fasted, were 1.19 (1.13, 1.25), 1.18 (1.13, 1.25), and 1.03 (0.89, 1.18), respectively (Table 3 ). All GMRs and 90% CIs in the fed and fasted states were contained within the bioequivalence bounds of 0.80 and 1.25, indicating that molnupiravir F1 tablets can be administered without regard to the timing of food intake. Other PK parameter estimates were generally similar between fed and fasted states, apart from t_1/2_, which was numerically higher in the fed state (Table 3). Molnupiravir was rapidly absorbed and converted to NHC in both the fed and fasted states following administration of the F1 tablet, with a median T_max_ (range) of 1.5 hours (1.00–2.00 hours) and 1.5 hours (1.00–4.00 hours), respectively (Table 3). The mean concentration-time profiles of both fed and fasted states were similar (Fig. 3).

**TABLE 3:** Statistical comparison and summary statistics of NHC plasma pharmacokinetics following the administration of one 400 mg molnupiravir Formulation 1 tablet in the fasted state (Treatment B) and in the fed state (Treatment C) in healthy adults^*a*^

Pharmacokinetic parameter	One 400 mg molnupiravir Formulation 1 tablet (fasted state)			One 400 mg molnupiravir Formulation 1 tablet (fed state)			Ratio^*e*^		% CV^*f*^
	N	GM	95% CI	N	GM	95% CI	GMR	90% CI	
AUC_0–inf_ (hr⋅ng/mL)^*b*^	16	4,250	(3,560, 4,940)	16	5,030	(4,540, 5,580)	1.19	(1.13, 1.25)	8.2
AUC_0–last_ (hr⋅ng/mL)^*b*^	16	4,240	(3,650, 4,930)	16	5,020	(4,530, 5,570)	1.18	(1.13, 1.25)	8.2
AUC_0–12_ (hr⋅ng/mL)^*b*^	16	4,200	(3,610, 4,880)	16	4,930	(4,470, 5,450)	1.17	(1.11, 1.24)	8.6
C_max_ (ng/mL)^*b*^	16	1,770	(1,480, 2,120)	16	1,820	(1,620, 2,040)	1.03	(0.89, 1.18)	22.7
T_max_ (hr)^*c*^	16	1.50	(1.00, 2.00)	16	1.50	(1.00, 4.00)	NC	NC	NC
t_½_ (hr)^*d*^	16	3.23	67.9	16	5.03	88.7			
CL/F (L/hr)^*d*^	16	74.2	28.9	16	62.5	19.5			
V_z_/F (L/hr)^*d*^	16	346	62.5	16	454	84.8			

^*a*^ AUC_0–12_, area under the concentration-time curve from time 0 to 12 hours; AUC_0–inf_, area under the concentration-time curve from time 0 to infinity; AUC_0–last_, area under the concentration-time curve from time 0 to the last measurable time point; CI, confidence interval; CL/F, apparent plasma clearance; C_max_, maximum plasma concentration; CV, coefficient of variation; GM, geometric mean; GMR, geometric mean ratio; NC, not calculated; NHC, β-D-N4-hydroxycytidine; t_1/2_, half-life; T_max_, time to maximum plasma concentration; Vz/F, apparent volume of distribution.

^*b*^ Back-transformed least squares mean (mean difference) and CI from linear mixed-effects model performed on natural log-transformed values.

^*c*^ Median (min, max) reported for T_max_.

^*d*^ Geometric mean and percent geometric CV reported for t_½_, CL/F, and V_z_/F.

^*e*^ Reported as one 400 mg molnupiravir Formulation 1 tablet (fed state)/one 400 mg molnupiravir Formulation 1 tablet (fasted state).

^*f*^ Pseudo within-subject % CV = 100 × Sqrt[(σp2 + σq2 – 2 × σpq)/2], where σp2 and σq2 are the estimated variances on the log scale for the two treatment groups, and σpq is the corresponding estimated covariance, each obtained from the linear mixed-effects model.

To determine if the rate of dissolution of the molnupiravir tablet formulation *in vitro* affects the clinical PK of NHC, PK parameter estimates were compared between the molnupiravir reference capsule formulation and the molnupiravir F2 tablet (Table 4). The NHC AUC_0–inf_, AUC_0–last_, and C_max_ GMRs (90% CIs), reported as molnupiravir F2 tablet/reference capsule, were 1.05 (1.01, 1.09), 1.05 (1.01, 1.09), and 0.96 (0.90, 1.03), respectively (Table 4). While not prespecified, the 90% CIs for the GMRs of plasma NHC PK parameters were within the standard bioequivalence bounds of 0.80 and 1.25, demonstrating the similarity of the molnupiravir F2 tablet and molnupiravir reference capsules. Other PK parameters were also generally similar, with only numeric differences observed between the two formulations. The absorption profile of NHC was generally similar, with a median T_max_ (range) of 1.5 hours (1.00–3.00 hours) following administration of both formulations.

**TABLE 4:** Statistical comparison and summary statistics of NHC plasma pharmacokinetics following the administration of two 200 mg molnupiravir reference capsules in the fasted state (Treatment A) and one 400 mg molnupiravir Formulation 2 tablet in the fasted state (Treatment D) in healthy adults^*a*^^,^^*b*^

Pharmacokinetic parameter	Two 200 mg molnupiravir reference capsules (fasted state)			One 400 mg molnupiravir Formulation 2 tablet (fasted state)				Ratio^*f*^	% CV^*g*^
	N	GM	95% CI	N	GM	95% CI	GMR	90% CI	
AUC_0–inf_ (hr⋅ng/mL)^*c*^	48	3,970	(3,680, 4,280)	47	4,160	(3,860, 4,490)	1.05	(1.01, 1.09)	11.9
AUC_0–last_ (hr⋅ng/mL)^*c*^	48	3,960	(3,680, 4,270)	47	4,160	(3,850, 4,480)	1.05	(1.01, 1.09)	11.9
AUC_0–12_ (hr⋅ng/mL)^*c*^	48	3,930	(3,650, 4,240)	47	4,110	(3,820, 4,480)	1.05	(1.00, 1.09)	11.9
C_max_ (ng/mL)^*c*^	48	1,660	(1,520, 1,810)	47	1,600	(1,470, 1,740)	0.96	(0.90, 1.03)	19.6
T_max_ (hr)^*d*^	48	1.5	(1.00, 3.00)	47	1.5	(1.00, 3.00)	NC	NC	NC
t_½_ (hr)^*e*^	48	2.76	69.5	47	3.69	94.3			
CL/F (L/hr)^*e*^	48	79.3	26.0	47	75.4	26.5			
Vz/F (L/hr)^*e*^	48	315	65.9	47	402	80.2			

^*a*^ AUC_0–12_, area under the concentration-time curve from time 0 to 12 hours; AUC_0–inf_, area under the concentration-time curve from time 0 to infinity; AUC_0–last_, area under the concentration-time curve from time 0 to the last measurable time point; CI, confidence interval; CL/F, apparent plasma clearance; C_max_, maximum plasma concentration; CV, coefficient of variation; GM, geometric mean; GMR, geometric mean ratio; NC, not calculated; NHC, β-D-N4-hydroxycytidine; t_1/2_, half-life; T_max_, time to maximum plasma concentration; Vz/F, apparent volume of distribution.

^*b*^ One participant did not participate in Period 3.

^*c*^ Back -transformed least squares mean (mean difference) and confidence intervalCI from linear mixed-effects model performed on natural log-transformed values.

^*d*^ Median (min, max) reported for Tmax.

^*e*^ Geometric mean and percent geometric CV reported for t½, CL/F, and Vz/F.

^*f*^ Reported as one 400- mg molnupiravir Formulation 2 tablet (fasted state) / two 200- mg molnupiravir reference capsules.

^*g*^ Pseudo within-subject % CV = 100 × Sqrt [(σp2 + σq2 - 2 × σpq)/2], where σp2 and σq2 are the estimated variances on the log scale for the 2two treatment groups, and σpq is the corresponding estimated covariance, each obtained from the linear mixed-effects model.

### Safety

Molnupiravir was found to be generally well tolerated as a capsule or tablet formulation in this study. One participant discontinued from the study on day 5 after completing Period 1 (Treatment A) due to an abnormal electrocardiogram (ECG) finding of type 3 Brugada pattern (asymptomatic), which was detected on scheduled safety monitoring and was deemed as not related to study drug by the investigator. Overall, following the administration of molnupiravir (inclusive of all dosage formulations, with or without food), 18.8% of participants (*n* = 12/64) experienced one or more adverse events (AEs), the most frequently reported of which (i.e., those experienced by ≥2 participants in total) were headache (*n* = 4; 6.3%), cough (*n* = 4; 6.3%), and constipation (*n* = 3; 4.7%) (see Table S1 for the full listing). All AEs were mild in intensity, and there was no apparent relationship between reported AEs and treatment (i.e., molnupiravir formulation, molnupiravir dosage, and feeding status). Overall, four participants experienced drug-related AEs. No serious AEs were reported, and none of the participants discontinued from the study due to a drug-related AE. There were no other clinically meaningful findings (whether reported as an AE or not) in laboratory evaluation, vital signs, or ECG parameter values in the study.

## DISCUSSION

In this randomized, open-label, four-treatment sequence, three-period crossover study, a single oral 400 mg dose of the newly developed film-coated tablet formulation of molnupiravir (F1) was found to be bioequivalent to the currently authorized reference capsule formulation. The primary hypothesis of bioequivalence was supported; the 90% CIs of the GMRs for plasma NHC AUC_0–inf_, AUC_0–last_, and C_max_ (reported as F1 tablet/reference capsule) were contained within the prespecified bounds of 0.80 to 1.25.

There was no meaningful effect of food on the absorption or bioavailability of the molnupiravir F1 tablet, as demonstrated by the similarity of PK parameter estimates (AUC_0–inf_, AUC_0–last_, T_max_, and C_max_) in fed and fasted states, indicating that the molnupiravir F1 tablet may be administered without regard to food. The half-life of NHC was estimated to be higher following administration in the fed state; however, estimation of this parameter was associated with high variability because most of the concentrations in the terminal phase of the concentration-time profile were below the lower limit of quantitation (LLOQ) in this study. Overall, the magnitude of the difference in half-life was small and not clinically relevant, as demonstrated by the similarity for other PK parameters (Tables 2 and 3).

We observed that NHC rapidly reached peak concentrations at 1.5 hours postdose, in line with previous phase 1 data on the PK of molnupiravir (15). The PK of NHC, the primary circulating analyte, was measured in this study rather than the prodrug molnupiravir, as the prodrug is generally not detectable in the plasma at the dose administered in this study (400 mg) (2). These data indicate high intestinal absorption for both molnupiravir and NHC, results that are in line with observations from preclinical PK studies, in which the bioavailability of NHC after oral administration of molnupiravir was 52% in rats and ≥77% in dogs (16). Taken together, these observations suggest that due to its high solubility and permeability, molnupiravir resembles a Biopharmaceutics Classification System (BCS) Class I drug (17).

The plasma PK of NHC following oral administration of a slower-release molnupiravir tablet (F2) was compared with the F1 immediate-release tablet. The two tablet formulations are qualitatively similar, but the F2 tablet has a slower *in vitro* dissolution profile than the F1 tablet. The molnupiravir F2 tablet was designed to be near the lower limit of bioequivalence with the molnupiravir reference capsules, while the molnupiravir F1 tablet had an *in vitro* dissolution profile predicting bioequivalence to the molnupiravir reference capsules. The molnupiravir F2 tablet was included in this study to define and widen the dissolution bioequivalence safe space with clinical data, as well as to better understand the PK performance of the drug product (11, 12). The absorption and bioavailability of the molnupiravir F2 tablet were found to be similar to the reference capsule formulation, and therefore, also similar to the molnupiravir F1 tablet.

In this study, molnupiravir was generally well tolerated as both capsule and tablet formulation in healthy adults, with or without food, with a safety profile similar to that previously reported in other molnupiravir clinical studies, in which 18.8% of patients experienced one or more AEs, all of which were mild in intensity (2, 15, 18–23). No serious AEs were reported. The most commonly reported AEs were headache and cough. No drug-related AEs led to early discontinuation of study intervention. The single participant who was discontinued from the study (after completing Period 1) was discontinued due to an abnormal ECG with type 3 Brugada pattern (asymptomatic), which was detected during protocol-scheduled safety monitoring and was not considered related to study intervention.

One potential limitation of this study was the narrow range of weights and ethnicities of the enrolled study population. However, weight and ethnicity are not expected to impact the extent of absorption of molnupiravir, and the results from this study should be broadly applicable (24). Additionally, this study was strengthened by a relatively large sample size (*N* = 64) compared with other bioequivalence studies, which often enroll between 24 and 36 participants (25).

In conclusion, the primary hypothesis of bioequivalence between a 400 mg oral dose of molnupiravir reference capsules (administered as two 200 mg capsules) and a single 400 mg oral dose of an immediate-release molnupiravir F1 tablet was met. Food did not meaningfully impact the rate or extent of absorption of the F1 tablet. Molnupiravir was generally well tolerated in both capsule and tablet formulations in this healthy adult study population. The demonstration of bioequivalence between the tablet and capsule formulations enables a commercial transition to a formulation that reduces pill burden, which is particularly relevant for patients who have difficulty swallowing or already have a high pill burden of other medications because of underlying comorbidities. The unique study design enabled the opportunistic evaluation of a second molnupiravir F2 tablet formulation within a traditional bioequivalence study. Such a study design may be considered to simultaneously establish a bioequivalent formulation for commercialization while expanding the dissolution bioequivalence safe space, which can aid in understanding the PK performance of the drug product and support drug product quality (11).

## MATERIALS AND METHODS

### Study design

This randomized, open-label, three-period crossover study was performed at a single, nonhospital clinical research site in the United States. The study was conducted in accordance with principles of Good Clinical Practice and was approved by the appropriate institutional review boards and regulatory agencies. All participants provided written informed consent prior to enrollment. This study was registered at ClinicalTrials.gov as NCT06615869.

A total of 64 eligible participants were randomly assigned 1:1:3:3 into four open-label treatment sequences using a computer-generated allocation schedule for Periods 1, 2, and 3 (Fig. 1). Specifically, participants were assigned to the treatment sequences: (i) A-B-C, *n* = 8; (ii) B-A-C, *n* = 8; (iii) A-B-D, *n* = 24; and (iv) B-A-D, *n* = 24. The study consisted of three sequential study periods. Periods 1 and 2 consisted of two treatments to evaluate the definitive bioequivalence of molnupiravir reference capsule formulation (two 200 mg capsules, Treatment A) and molnupiravir F1 tablet (one 400 mg tablet, Treatment B). Before Period 1, each participant was randomly assigned 1:1 to initiate either Treatment A or B at the onset of Period 1, and 1:3 to were randomly assigned to either Treatment C or D in Period 3. The 1:3 ratio for Treatment C versus D was selected to ensure a robust evaluation of the food effect on PK, namely, *n* = 16 to ensure at least *n* = 12 completed Treatment C, based on the observation that there was no meaningful effect of food on the PK of the reference formulation (2) and FDA Food-Effect Study Guidance (26). The larger proportion of participants (*n* = 48) was assigned to Treatment D to enable rigorous PK comparison of the F1 and F2 formulations given that the PK profiles were expected to be on the edge of bioequivalence. All participants (*N* = 64) received either Treatment A or B in Period 1, and then crossed over to receive the other treatment in Period 2, as per the 1:1 randomization at study onset (*n* = 32 for each sequence of Treatments A and B). All doses were administered in the fasted state, and there was at least a 4-day washout period between dosing in each treatment period. Following completion of Periods 1 and 2, participants entered Period 3, consisting of two potential treatments: Treatment C (single-dose administration of one 400 mg molnupiravir F1 tablet in the fed state *n* = 16) to evaluate the effect of food on the plasma NHC PK with the molnupiravir F1 tablet or Treatment D (one 400 mg molnupiravir F2 tablet; *n* = 48) to assess the relative bioavailability of the molnupiravir F2 tablet compared with the reference capsule formulation.

Study intervention(s) were administered in the clinical research unit, by the investigator and/or study staff, on the morning of day 1 of Periods 1, 2, and 3, following an overnight fast of at least 10 hours, except for Treatment C in Period 3. Treatment C was administered approximately 30 minutes after having a standard, high-fat breakfast. There was at least a 4-day washout period between treatment periods.

Blood samples were drawn to determine NHC concentrations in plasma at predose and at selected time points (0.5, 1, 1.5, 2, 3, 4, 5, 6, 8, 12, 24, and 48 hours) up to 72 hours following the dose. Unless a participant remained domiciled in the clinic at the 48- and/or 72-hour time points following the dose, there were additional outpatient visits on day 3 and day 4 of Periods 2 and 3 following the dose, for the collection of the 48- and 72-hour samples following the dose. A follow-up visit occurred approximately 14 days after the dose to review prior/concomitant medications and AEs/SAEs and to measure serum human gonadotropin (in women of childbearing potential [WOCBP] only) and vital signs.

### Study population

Healthy male or female participants between the ages of 18 and 60 years (inclusive) and with a body mass index between 18 and 32 kg/m^2^ (inclusive) were eligible to participate in this study. Male participants were eligible for inclusion if they agreed to, during the intervention period and for at least 90 days after the last dose of the study intervention, abstain from heterosexual intercourse or use contraception. Female participants were eligible for inclusion if they were not pregnant or breastfeeding, not a WOCBP, or a WOCBP and using a highly effective contraceptive method. Potential participants were screened for inclusion in the study within 28 days of the first dose administration after voluntarily providing informed consent.

### Pharmacokinetic measurements and methods

Plasma NHC concentrations were collected and analyzed by Labcorp Early Development Laboratories Inc. (Madison, WI, USA) using a validated liquid chromatography with tandem mass spectrometry (LC-MS/MS) method previously described (27). The analytical method used protein precipitation for analyte isolation, followed by LC-MS/MS detection. The LLOQ was 1.00 ng/mL, with a linear calibration range from 1.00 to 1000 ng/mL (upper limit of quantitation).

The plasma NHC concentration profiles were used to calculate the following PK end points: AUC_0–inf_, AUC_0–last_, AUC from time 0 to 12 hours (AUC_0–12_), C_max_, T_max_, t_½_, estimated drug for oral clearance (CL/F), and estimated volume of distribution for oral dose (V_z_/F) of NHC. Plasma PK parameters were calculated by noncompartmental analyses using the software Phoenix WinNonlin Professional (Version 8.1; Certara USA, Inc., Princeton, USA). C_max_ and T_max_ were generated from the observed plasma concentration-time data. AUC was calculated using the linear trapezoidal method for ascending concentrations and the log trapezoidal method for descending concentrations (linear-up/log-down). AUC_0–inf_ was calculated as the sum of AUC_0–last_ plus the estimated concentration corresponding to the time of the last measurable concentration (C_est,last_)/λ. For each participant, λ was calculated by regression of the terminal log-linear portion of the plasma concentration-time profile; at least three data points (excluding the C_max_) in the terminal log-linear phase were used for λ calculations. The onset of the terminal log-linear phase was determined using the Phoenix WinNonlin Auto Selection “Best Fit” method and reviewed for reasonableness by the pharmacokineticist. The apparent terminal t_½_ was calculated as the quotient of the natural log of 2 (ln [2]) and λ. CL/F was calculated as the quotient of dose and AUC_0–inf_. V_z_/F was calculated as the quotient of CL/F and λz.

### Safety assessments

Safety was monitored throughout the study by repeated clinical and laboratory evaluations, including AEs, laboratory safety tests (serum chemistry, hematology, and urinalysis), ECG, and vital signs (body temperature, heart rate, respiratory rate, and blood pressure). AEs, serious AEs, and other reportable safety events that occurred after the participant provided documented informed consent, but before intervention randomization, were reported by the investigator to the study sponsor if the event caused the participant to be excluded from the study, or if it was the result of a protocol-specified intervention.

### Statistical analysis

Descriptive statistics were used to summarize the safety data. All statistical analyses were conducted using SAS software (Version 9.4; SAS Institute Inc., Cary, NC, USA). In Periods 1 and 2, separately for each abovementioned PK end point, individual values of AUC_0–inf_, AUC_0–last_, AUC_0–12_, and C_max_ for each of the treatments (molnupiravir F1 tablets and molnupiravir reference capsules) were natural log-transformed and were evaluated using a linear mixed-effects model, with fixed effect for treatment and period. An unstructured covariance matrix was used to allow for unequal treatment variances and to model the correlation between different treatment measurements within the same participant via the REPEATED statement in SAS PROC MIXED. The Kenward-Roger method was used to calculate the denominator degrees of freedom for the fixed effects (DDFM = KR) (28). For each of the aforementioned PK end points, the least squares mean differences and two-sided 90% CIs (molnupiravir F1 tablet–molnupiravir reference capsules) on the natural log scale were calculated. The least squares mean differences and CIs were exponentiated to obtain GMRs and 90% CIs for the true ratios (molnupiravir F1 tablet/molnupiravir reference capsules). Bioequivalence between the molnupiravir reference capsule (administered as two 200 mg capsules) and the molnupiravir F1 tablet (administered as one 400 mg tablet) was concluded if the 90% CIs for the true GMRs (F1 tablet/reference capsule) for plasma NHC AUC_0–inf_, AUC_0–last_, and C_max_ were all contained within the range of 0.80 to 1.25, in accordance with the FDA draft guidance for bioequivalence and the EMA guidance for bioequivalence (13, 14).

In Period 3, to estimate the effect of food on the plasma PK of NHC following administration of the molnupiravir F1 tablet, individual values of AUC_0–inf_, AUC_0–last_, AUC_0–12_, and C_max_ from Treatments B and C were natural log-transformed and were evaluated using a linear mixed-effects model, with a fixed effect for treatment. GMRs (F1 with food/F1 with water) and 90% CIs were estimated as described above. An unstructured covariance matrix was used to account for unequal treatment and interparticipant variability as described above. Bioequivalence in the fed and fasted state was concluded if the 90% CIs for the true GMRs (F1 with food/F1 with water) for plasma NHC AUC_0–inf_, AUC_0–last_, and C_max_ were all contained within 0.80 and 1.25, as per the FDA clinical pharmacology considerations for assessing the effects of food on drugs for orally administered drug products (29).

To estimate the relative bioavailability of the molnupiravir F2 tablet, individual values of AUC_0–inf_, AUC_0–last_, AUC_0–12_, and C_max_ from Treatments D and A were natural log-transformed and evaluated using a linear mixed-effects model with a fixed effect for treatment. GMRs (molnupiravir F2 tablet/molnupiravir reference capsules) and 90% CIs were estimated as described previously. An unstructured covariance matrix was used to account for unequal treatment and interparticipant variability, also as described previously. Bioequivalence was concluded if the 90% CIs for the true GMRs (F2 tablet/reference capsule) for plasma NHC AUC_0–inf_, AUC_0–last_, and C_max_ were all contained within bioequivalence intervals (0.80, 1.25), in accordance with the FDA draft guidance for bioequivalence and the EMA guidance for bioequivalence (13, 14).

## Data Availability

The data sharing policy, including restrictions, of Merck Sharp & Dohme LLC, a subsidiary of Merck & Co., Inc., Rahway, NJ, USA (MSD), is available at https://trialstransparency.msdclinicaltrials.com/policies-perspectives.aspx. Requests for access to the clinical study data can be submitted via email to dataaccess@msd.com.

## SUPPLEMENTAL MATERIAL

Table S1 aac.01434-24-s0001.docx Adverse events (regardless of relationship to study intervention) in the safety population.

ASM does not own the copyrights to Supplemental Material that may be linked to, or accessed through, an article. The authors have granted ASM a non-exclusive, world-wide license to publish the Supplemental Material files. Please contact the corresponding author directly for reuse.
