# Supplementary material for: Pharmacokinetics and bioequivalence of a molnupiravir tablet formulation compared with the molnupiravir capsule formulation in healthy adult participants—a randomized, open-label, three-period, crossover study
Source: Antimicrob Agents Chemother. 2025 Feb 6;69(3):e01434-24. doi: 10.1128/aac.01434-24 (PMC11881578; doi:10.1128/aac.01434-24)
Supplement: Table S1 — Adverse events (regardless of relationship to study intervention) in the safety population. [file aac.01434-24-s0001.docx]

**Supplementary Materials**

**Table S1. Listing of adverse events (regardless of relationship to study intervention) in the safety population.**

|  | | **Treatment A** | | | **Treatment B** | | | **Treatment C** | | | **Treatment D** | | **Total** | | |  |
| --- | --- | --- | --- | --- | --- | --- | --- | --- | --- | --- | --- | --- | --- | --- | --- | --- |
|  | n | | (%) | n | | (%) | n | | (%) | n | | (%) | | n | (%) | |
| Participants in population | 64 | |  | 63 | |  | 16 | |  | 47 | |  | | 64 |  | |
| With 1 or more adverse events | 7 | | (10.9) | 6 | | (9.5) | 1 | | (6.3) | 3 | | (6.4) | | 12 | (18.8) | |
| With no adverse events | 57 | | (89.10) | 57 | | (90.5) | 15 | | (93.8) | 44 | | (93.6) | | 52 | (81.3) | |
| **Eye disorders** | **0** | | **(0.0)** | **1** | | **(1.6)** | **0** | | **(0.0)** | **1** | | **(2.1)** | | **1** | **(1.6)** | |
| Eyelid edema | 0 | | (0.0) | 1 | | (1.6) | 0 | | (0.0) | 1 | | (2.1) | | 1 | (1.6) | |
| **Gastrointestinal disorders** | **1** | | **(1.6)** | **3** | | **(4.8)** | **0** | | **(0.0)** | **0** | | **(0.0)** | | **4** | **(6.3)** | |
| Constipation | 1 | | (1.6) | 2 | | (3.2) | 0 | | (0.0) | 0 | | (0.0) | | 3 | (4.7) | |
| Nausea | 0 | | (0.0) | 1 | | (1.6) | 0 | | (0.0) | 0 | | (0.0) | | 1 | (1.6) | |
| **Infections and infestations** | **2** | | **(3.1)** | **0** | | **(0.0)** | **0** | | **(0.0)** | **0** | | **(0.0)** | | **2** | **(3.1)** | |
| Conjunctivitis | 1 | | (1.6) | 0 | | (0.0) | 0 | | (0.0) | 0 | | (0.0) | | 1 | (1.6) | |
| Upper respiratory tract infection | 1 | | (1.6) | 0 | | (0.0) | 0 | | (0.0) | 0 | | (0.0) | | 1 | (1.6) | |
| **Injury, poisoning and**  **procedural complications** | **1** | | **(1.6)** | **0** | | **(0.0)** | **0** | | **(0.0)** | **0** | | **(0.0)** | | **1** | **(1.6)** | |
| Fall | 1 | | (1.6) | 0 | | (0.0) | 0 | | (0.0) | 0 | | (0.0) | | 1 | (1.6) | |
| **Investigations** | **1** | | **(1.6)** | **0** | | **(0.0)** | **0** | | **(0.0)** | **0** | | **(0.0)** | | **1** | **(1.6)** | |
| Electrocardiogram abnormality^a^ | 1 | | (1.6) | 0 | | (0.0) | 0 | | (0.0) | 0 | | (0.0) | | 1 | (1.6) | |
| **Musculoskeletal and**  **connective tissue disorders** | **1** | | **(1.6)** | **1** | | **(1.6)** | **0** | | **(0.0)** | **0** | | **(0.0)** | | **1** | **(1.6)** | |
| Arthralgia | 1 | | (1.6) | 0 | | (0.0) | 0 | | (0.0) | 0 | | (0.0) | | 1 | (1.6) | |
| Neck pain | 0 | | (0.0) | 1 | | (1.6) | 0 | | (0.0) | 0 | | (0.0) | | 1 | (1.6) | |
| **Nervous system disorders** | **1** | | **(1.6)** | **0** | | **(0.0)** | **1** | | **(6.3)** | **2** | | **(4.3)** | | **4** | **(6.3)** | |
| Headache | 1 | | (1.6) | 0 | | (0.0) | 1 | | (6.3) | 2 | | (4.3) | | 4 | (6.3) | |
| **Respiratory, thoracic and mediastinal disorders** | **2** | | **(3.1)** | **2** | | **(3.2)** | **0** | | **(0.0)** | **0** | | **(0.0)** | | **4** | **(6.3)** | |
| Cough | 2 | | (3.1) | 2 | | (3.2) | 0 | | (0.0) | 0 | | (0.0) | | 4 | (6.3) | |
| **Vascular disorders** | **0** | | **(0.0)** | **1** | | **(1.6)** | **0** | | **(0.0)** | **0** | | **(0.0)** | | **1** | **(1.6)** | |
| Phlebitis | 0 | | (0.0) | 1 | | (1.6) | 0 | | (0.0) | 0 | | (0.0) | | 1 | (1.6) | |
| Every participant is counted a single time for each applicable row and column.  ^a^ The adverse event was as an electrocardiogram finding of a type 3 Brugada pattern. The participant was asymptomatic. Diagnosis of Brugada syndrome requires further clinical subspecialty (cardiology) evaluation.  Treatment A = Two 200-mg molnupiravir reference capsules (fasted state)  Treatment B = One 400-mg molnupiravir Formulation 1 tablet (fasted state)  Treatment C = One 400-mg molnupiravir Formulation 1 tablet (fed state)  Treatment D = One 400-mg molnupiravir Formulation 2 tablet (fasted state) | | | | | | | | | | | | | | | |  |
